# Establishing an oncocardiology service

**DOI:** 10.1007/s00059-020-04952-w

**Published:** 2020-06-22

**Authors:** L. H. Lehmann, M. Totzeck

**Affiliations:** 1 Department of Cardiology, Angiology, Pneumology, Cardio-Oncology Unit, Heidelberg University Hospital, Im Neuenheimer Feld 410, 69120 Heidelberg, Germany; 2 DZHK (German Centre for Cardiovascular Research), partner site Heidelberg/Mannheim, Heidelberg, Germany; 3 Deutsches Krebsforschungszentrum (DKFZ), Heidelberg, Germany; 4 Department of Cardiology and Vascular Medicine, West German Heart and Vascular Center, University Hospital Essen, Medical Faculty, University of Duisburg-Essen, Essen, Germany

**Keywords:** Cardiotoxicity, Heart failure, Comorbidities, Arrhythmia, Cardio-oncology, Kardiotoxizität, Herzinsuffizienz, Komorbiditäten, Arrhythmie, Kardioonkologie

## Abstract

Oncocardiology is an emerging field in cardiovascular healthcare. Besides establishing surveillance and follow-up strategies for cancer patients, it will be essential to set up specialized oncocardiology services. However, there is a lack of clinical studies to give evidence-based recommendations regarding cardiological diagnostic and therapeutic approaches for cancer patients. An oncocardiology service is a patient-centered structure that aims to integrate research and interdisciplinary patient care to bridge this gap. We discuss the current challenges in developing an oncocardiology service and review the literature on this topic. We further provide an overview of the essential diagnostic tools and upcoming ethical issues to be considered in the management of oncology patients.

## Abstract (de)

Die Onkokardiologie ist ein neuer, aufstrebender Bereich in der kardiovaskulären Gesundheitsversorgung. Neben der Etablierung von Monitoring- und Nachsorgestrategien für onkologische Patienten ist es wichtig, in Zukunft ein Netzwerk spezialisierter onkokardiologischer Spezialambulanzen zu errichten. Aktuell sind nur wenige prospektive klinische Studien verfügbar, die eine evidenzbasierte Empfehlung für kardiologische Diagnose- und Therapieansätze bei onkologischen Patienten geben könnten. Eine onkokardiologische Ambulanz ist eine patientenzentrierte Struktur, die darauf abzielt, Forschung und interdisziplinäre Patientenversorgung zu integrieren, um diese Lücke vorerst zu schließen. Die Autoren diskutieren die Herausforderungen bei der Entwicklung einer Spezialambulanz für Onkokardiologie unter Berücksichtigung der aktuellen Literatur zu diesem Thema. Darüber hinaus wird ein Überblick über die wesentlichen diagnostischen Instrumente und ethische Abwägungen bei onkologischen Patienten gegeben.

## The heart matters

Advances in cancer treatment and “personalized therapy” strategies have improved the survival of many cancer patients. As a consequence, cancer comorbidities such as cardiac disease are of growing importance for cancer patients. Moreover, cardiac pathologies are reported to play a more relevant role in morbidity and mortality of several conditions than the underlying malignant disease itself [1–3].

In addition to common risk factors for cardiac disease and cancer, the direct effects of tumors and their therapies can lead to cardiac pathologies. To reduce mortality and morbidity and to improve patient care, it will be essential to establish a network of oncocardiology services that allows us to enhance cardiac surveillance strategies and to improve adherence to cardiological diagnostics and therapy. Registries as well as clinical and basic research should form the basis for further evidence-based strategies.

**Fig. 1:**
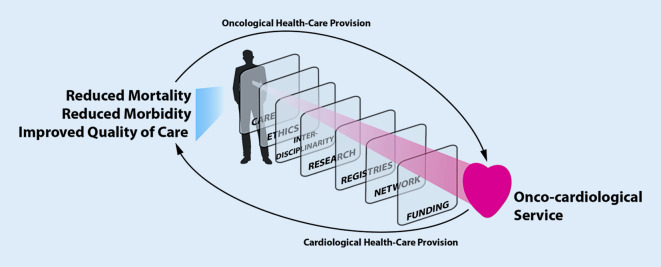
An oncocardiology service is the interdisciplinary integration of cardiological and oncological strategies. Permanent integration of cardiological healthcare into oncological healthcare will help to reduce mortality and morbidity and to improve quality of care. Multiple challenges need to be overcome to set up this structure

However, the patient-centered integration of all layers of an oncocardiology service is a challenge (Fig. 1).

## Local requirements

Establishing an oncocardiology service depends on the local requirements. It is crucial to identify the patients who will potentially benefit from this service. However, the oncocardiology service needs to be adapted to already-established follow-up strategies. For example, patients with breast cancer and presumed long-term survival will require a different setting than patients with hematological diseases who will undergo transplantation in the near future or patients with gastroesophageal cancers with neoadjuvant therapies followed by surgical intervention. Starting with a circumscribed patient cohort simplifies the creation of an oncocardiology service.

The European Society of Cardiology (ESC) distinguishes between an oncocardiology service that is applicable to general/district hospitals, tertiary hospitals, or selected centers [4]. The main difference is the number and diversity of patients who are potentially admitted to the service.

**Fig. 2:**
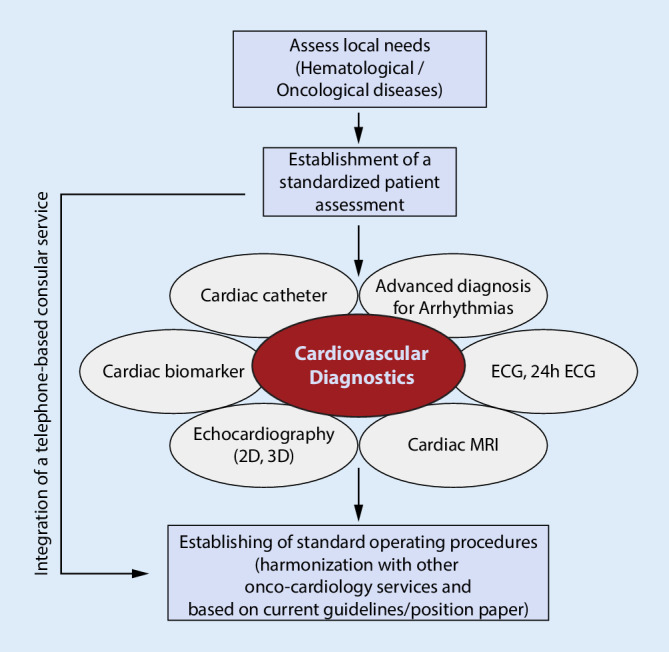
Adaptation to local needs and integration of diverse cardiac diagnostic needs are necessary. For early stratification of patients, an additional telephone-based consultation service might be helpful for larger clinical centers. *MRI* magnetic resonance imaging

In university or large nonacademic hospitals, the number of patients who potentially need oncocardiological assessment is high. Therefore, a telephone-based consultation service might be helpful for the pre-assessment of patients (Fig. 2).

## Who needs an oncocardiology service?

Besides the “classic” cardiotoxic cancer drugs, such as *anthracyclines* or *HER2 antibodies*, there is a growing number of novel classes of therapeutics. Among these novel therapies, *immune checkpoint inhibitors, proteasome inhibitors, BRAF inhibitors,* and other *kinase inhibitors* are particularly associated with cardiotoxic effects. Patients for whom these potential cardiotoxic therapies are planned should be assessed by a cardiologist before, during, and after therapy.

Furthermore, patients with a high risk of cardiovascular disease (history of smoking, hypertension, reduced ejection fraction) also have a higher risk of cardiac side effects associated with oncological therapies. These patients should also be assessed if systemic therapy is needed.

A guide on which patients need cardiological assessment and how to monitor these patients was recently published as a position statement by the German Society of Cardiology (DGK; [5]).

Importantly, only very few data from prospective clinical trials are currently available and the mechanisms of cardiotoxicity are not well understood. This makes it particularly difficult to establish an “evidence-based” surveillance strategy for patients on cancer therapies. In addition to the need for more clinical data, basic science has to investigate both cardioprotective strategies along with cancer treatments, as reflected in more recently published studies [6–8]. We recently established an oncocardiology working group within the German Society of Cardiology (AG40, *Arbeitsgruppe für Onkologische Kardiologie*) to set up a communication platform to initiate registries and clinical studies. The working group has close interactions with basic science groups to investigate mechanisms of cardiotoxicity and cancer-dependent cardiac disease.

For clinical routine, it is essential to communicate clear follow-up strategies. Admission and follow-up of cancer patients need to be adapted to the local oncology standards and prevailing oncology entities. The simplified aim of an oncocardiology service is based on two key points: (1) to facilitate successful cancer therapy and (2) to reduce the burden of cardiovascular pathologies. On this basis, patient care must be balanced to give priority to cancer therapy or cardioprotection.

## When do we need an oncocardiology service?

### Before oncological therapy

According to the current position paper, patients with an increased risk of developing cardiotoxicity or with a need for potential cardiotoxic therapy require cardiological assessment [9]. This assessment includes evaluation of individual cardiac risk factors (diabetes mellitus, hypertension, hyperlipidemia, and smoking), physical examination, electrocardiography, and imaging of the left ventricular ejection fraction by two- or three-dimensional echocardiography. There are a number of novel drugs, such as BRAF inhibitors or immune checkpoint inhibitors, where surveillance strategies are not well established. These patients should be seen in the oncocardiology service according to the current position statements [5].

However, cardiological assessment has to be adjusted to the potential high number of patients. Close cooperation with the local resident cardiologists for early stratification of patients is essential. Together, long-term follow-up strategies need to be established.

### During oncological therapy

During ongoing oncological therapy, most patients are admitted with either cardiological symptoms or with a high risk of cardiotoxic events in the absence of pathological findings.

Procedures for therapy-related complications and further cardiac diagnostics at this stage are performed on the basis of the current guidelines for the diagnosis and treatment of cardiac disease (e.g., acute coronary syndrome, heart failure, arrhythmia; [10–12]). There are very few specific guidelines for cardiotoxic complications, such as checkpoint inhibitor-associated myocarditis [13, 14].

### For cancer survivors

**Fig. 3:**
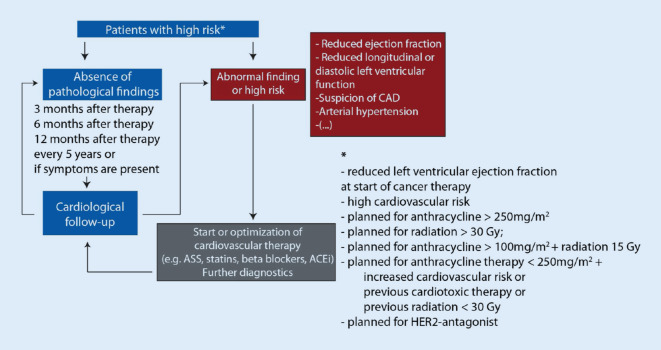
Exemplary, standardized follow-up strategy based on the current international guidelines and position paper as established in the onco-cardiology services in Heidelberg and Essen. Such flowcharts are starting points and should be adapted to the individual patient and can differ between cancer therapies. *CAD* coronary artery disease, *ACEi* angiotensin-converting enzyme inhibitors, *ASS* acetylsalicylic acid

Data on cardiological assessment after oncological therapies are mostly from patients who were successfully treated during childhood [15]. Again, patients at an increased risk of late cardiotoxicity need to be identified. Most data are available for patients after higher-dosage radiation and/or anthracycline-based therapies (Fig. 3). However, it is unclear whether novel cancer therapies also lead to late toxic events. It will be important to identify “high-risk” patients early, based on the individual predisposition. So far, we can only speculate on genomic, epigenomic, metabolic, and other risk-factors that may add to increased susceptibility for late toxicity. Ongoing research modeling cardiotoxicity on an individual level will be able to elucidate the mechanisms involved and provide insights for further diagnostic strategies [16].

## What do we need to assess?

There is strong evidence that patients with a high risk of cardiac disease also have an increased risk of developing cardiotoxic side effects [17]. Assessment of cardiac risk factors is therefore important in order to timely identify patients at risk, who need closer surveillance.

Many cancer therapies have the potential to induce arrhythmias. Therefore, baseline ECG and follow-up ECG are important for assessing cardiac rhythm, branch blocks, and QTc interval.

Most clinical decisions are made on the basis of left ventricular ejection fraction measurements with two- or three-dimensional echocardiography. Cardiac strain analysis is currently under discussion, but it might be helpful for the early detection of potential cardiotoxicity [18–21]. The best evidence of reduced strain as a potential marker for early cardiotoxicity is available from patients with breast cancer and anthracycline therapy.

Cardiac biomarkers such as troponin and N‑terminal pro-brain natriuretic peptide (NT-proBNP) are helpful for further stratifying patients during follow-up. Moreover, biomarker elevation can be an early sign of cardiotoxicity in many cancer therapies, whereas most of these data are from retrospective analysis of cancer studies [22, 23]. Prospective cohort studies are needed to improve the evidence for biomarker-based surveillance strategies.

## Future directions of oncocardiology diagnostics

Beside classic cardiac imaging and cardiac biomarkers, many potential novel diagnostic tools are currently being tested in the field of oncocardiology. Among them, positron emission tomography-computed tomography (PET-CT) to investigate cardiac metabolism and cardiac remodeling is the most promising [24]. Novel protocols for cardiac magnetic resonance imaging (cMRI) studies will also help in the timely identification of patients with potential cardiotoxicity [25].

## A multidisciplinary approach

The oncocardiology service should be embedded into a cardiology department, which includes availability of cardiac catheterization, myocardial biopsy, and advanced cardiac imaging [4].

For more complex side effects, as may be observed in patients on checkpoint inhibitors, an interdisciplinary team (cardiologist, neurologist, endocrinologist, gastroenterologist, oncologist) should be established.

## Are there guidelines available?

So far, there are only a limited number of clinical trials that fulfill criteria to allow for a clear recommendation on the open questions regarding the diagnosis, treatment, and surveillance of cancer patients. Currently, there are position papers available from the American Heart Association (AHA), the ESC [9], the American Society for Oncology (ASCO) together with the American College of Cardiology [21, 26, 27], and the European Society of Medical Oncology (ESMO; [28, 29]).

More recently, the German Society of Cardiology published a consensus paper on oncocardiology that includes all aspects of an oncocardiology service [5].

## Is there special training to run an oncocardiology service?

To run an oncocardiology service, it is essential to have a cardiologist with specific training in echocardiography who can perform and interpret physiological and pathological findings [21]. Furthermore, specific knowledge of the potential side effects of “classic” and novel cancer therapies is necessary so that advice can be given regarding future diagnostic needs. However, definitive decisions on the future direction of oncological therapies are based on team discussions, including the oncologist and the patient. Due to the large patient cohort and the increasing number of diverse cardiac side effects of oncological therapies, specific training for oncocardiology is considered necessary in large clinical centers [30–32]. The ESC Council on oncocardiology has launched specific webinars that are accredited by the European Accreditation Council for Continuing Medical Education (EACCME), which can be accessed on the ESC website. More recently, a board certificate was established by the International Society of Cardio-Oncology (ICOS), which was initiated to document specific expertise. Not only cardiologists but also oncologists, nurses, and physician assistants with an interest in cardiotoxicity are invited by the ICOS to complete the test for a certificate.

## Networking

Establishing an oncocardiology service involves tight interactions with the local oncology units. The oncocardiology service has to be adapted to the local needs based on the number of patients for certain cancer entities and therapies. Moreover, a close collaboration with the oncologist, e.g., in established tumor boards, is extremely helpful for implementing a cardiological perspective.

Due to the emerging need for clinical trials and further data from prospective studies, it will be crucial to build up a network of oncocardiologists. The growing interest in oncocardiology is also reflected by a number of international initiatives in oncological and cardiological societies and the establishment of specialized journals (e.g., *JACC: CardioOncology* and *Cardio-Oncology*). To allow a close interaction in Germany, we established a working group for oncocardiology within the German Society of Cardiology (DGK) and the German Society of Hematology and Clinical Oncology (DGHO). The primary goal is a strong collaboration between clinical and basic researchers. Together with oncologists, we are establishing standardized protocols to improve patient care so as to reduce cardiac morbidity and mortality [2, 5].

## Oncocardiology during a pandemic

With regard to the current situation of the global COVID-19 pandemic, oncocardiology services assess patients who have a high risk of mortality [33]. Most importantly, stratification of patients with a specific oncocardiological need is necessary in order to reduce patient contact. Integration of a telehealth system and a telephone-based consultation service (Fig. 2) will help reduce the total number of patients in daily routine [34].

## Ethical considerations

As mentioned earlier, an oncocardiology service aims to successfully treat cancer without harming the heart. The starting point for the patient is a life-threatening cancer. Frequently, decisions need to be made where the individual risk for cardiac toxicity needs to be accepted in order to successfully overcome cancer.

A clear therapeutic aim can help balance the patient’s individual priorities. So far, we can only speculate whether palliative patients might profit more from a cardioprotective strategy, whereas patients who can be potentially cured from cancer may instead accept the risk of cardiotoxicity. Naturally, for patients with a high chance of cure and with a presumed long-term survival, the risk of cardiotoxicity has to be reduced as much as possible.

Complex cardiological procedures also need to be discussed on an individual level. In many of the current cardiological guidelines, decisions are based on the survival time, which should be longer than 1 year (e.g., ICD implantation; [11]). However, patients on palliative care do not necessarily refuse complex cardiological procedures to avoid sudden death, and the desire for hastened death in patients on palliative care is low [35].

These few issues highlight the frequent ethical dilemmas that require careful contemplation and additional research in this field. The individual considerations regarding therapeutic strategies are based on team decisions including the cardiologist, the oncologist, and the patient.

## Conclusion

Thanks to several advances in cancer treatment, the survival of many cancer patients is improving. Consequently, cancer comorbidities such as cardiac disease are becoming increasingly important. Oncocardiology is an emerging field in cardiovascular healthcare that aims to facilitate successful cancer therapy and to reduce the burden of cardiovascular pathologies. An oncocardiology service is a patient-centered structure that integrates research as well as interdisciplinary patient care. Multiple challenges have to be overcome when setting up this new service, including organizational as well as ethical issues.
